# Stress Distribution Pattern in Mini Dental Implant-Assisted RPD with Different Clasp Designs: 3D Finite Element Analysis

**DOI:** 10.1155/2022/2416888

**Published:** 2022-03-11

**Authors:** Chaiy Rungsiyakull, Pimduen Rungsiyakull, Kullapop Suttiat, Nut Duangrattanaprathip

**Affiliations:** ^1^Department of Mechanical Engineering, Faculty of Engineering, Chiang Mai University Chiang Mai, Chiang Mai 50200, Thailand; ^2^Department of Prosthodontics, Faculty of Dentistry, Chiang Mai University, Chiang Mai 50200, Thailand; ^3^Prosthodontics Section, Department of Restorative Dentistry, Faculty of Dentistry, Naresuan University, Phitsanulok 65000, Thailand

## Abstract

**Introduction:**

The removable partial denture (RPD) components, especially the retentive arm, play a major role in the loading characteristic on supporting structures.

**Objective:**

To evaluate and compare the effect of different clasp designs on the stress distribution pattern, maximum von Mises stress, and average hydrostatic pressure on abutment teeth, as well as edentulous ridges, mini dental implants (MDIs), and peri-implant bone between the conventional removable partial denture (CRPD) and mini dental implant-assisted distal extension removable partial denture (IARPD) using a three-dimensional finite element analysis (3D FEA).

**Materials and Methods:**

3D FEA models of mandibular arches, with and without bilateral MDI at the second molar areas, and Kennedy class I RPD frameworks, with RPA, RPI, Akers, and no clasp component, were generated. A total of 200 N vertical load was bilaterally applied on both sides of distal extension areas, and the stress was analyzed by 3D FEA.

**Results:**

The stress concentration of IARPD with RPI clasp design was located more lingually on abutment teeth, MDI, and peri-implant bone, while the other designs were observed distally on the supporting structures. The maximum von Mises stress on the abutment root surface was decreased when the RPDs were assisted with MDIs. The CRPD and IARPD with the Akers clasp design showed the highest von Mises stress followed by the designs with RPA and RPI clasp, respectively. The average hydrostatic pressure in each group was in approximation.

**Conclusion:**

The placement of MDIs on distal extension ridges helps to reduce the stress concentration on denture supporting structures. The maximum von Mises stress is affected by the different designs of clasp components. The CRPD and the IARPD with RPI clasp provide the least stress on supporting structures.

## 1. Introduction

Among modern dental treatment modalities, the restoration of partial edentulous ridges with removable partial denture is accepted as a standard treatment option [1]. However, the compromise in denture retention and stability, especially in the mandible with distal extension base, is the most common clinical drawback for many patients [2, 3]. The difference in bearing capacities of the supporting tissues in the distal extension arch leads to the classic disadvantage of a removable partial denture, moving the denture and abutment torquing during function [4]. To overcome this inherent problem, functional impression technique has been applied to record the tissue in functional form as it reduces torque on supporting structures due to the difference in resiliency between the abutment teeth and soft tissue covered on edentulous ridges [5–7].

Clasp assembly design is the other promising strategy for altering the load distribution on the supporting structures [8–12]. The retentive component design based on stress breaker concept such as RPI or RPA clasp has been recommended by many clinicians for distal extension case. The disengaging of retentive tip from the abutment teeth following the downward movement of the distal extension base during the vertical occlusal loading found in this type of clasp design helps to neutralize the effect of noncompatible resiliency between abutment teeth and soft tissue covered on edentulous ridges, which results in reducing the load and torque on supporting structures [11, 13].

Nowadays, the placement of normal-sized implants distally to the free-end edentulous space, especially in the lower jaw to convert the Kennedy class I edentulous ridge into a pseudo class III, has been recommended as the other option to control the noncompatible resiliency between abutment teeth and soft tissue on distal extension ridges. The complementary support from dental implants placed on the edentulous ridge increases the stability and retention of the denture. In addition, it also reduces the stress loaded on supporting structures, which has resulted in decreasing traumas on those supporting tissues [2, 14–18].

However, the installation of normal-sized dental implants on edentulous ridges may not be possible for some patients for various reasons including anatomical limitations. To overcome this problem, the dental implant with smaller diameter called mini dental implants (MDIs) has been developed and applied. The success of using MDIs to gain the stability and retention of overdentures in patients who had compromised supporting bone has been addressed in previous studies [19–23]. However, Holmgren [24] found that the decrease in the bone-implant interface in case of MDIs resulted in higher and wider stress distribution in the peri-implant bone. This condition contributes to the decreasing ability of MDIs to carry or support the prostheses compared with the normal-sized dental implant. According to this drawback, the RPDs with mini dental implants assisted on the edentulous ridges should be concerned on controlling of torque loaded on supporting structures especially at the RPD abutment teeth and other supporting structures during functions. Therefore, the RPD clasp assembly design that minimizes torque on supporting structures could be concerned as another significant strategy for controlling the torque and load applying on supporting structures, especially in case of the distal extension base RPD assisted with mini dental implants.

From the literature review, there are few articles focused on the relationship between the RPD framework design and stress or torque loaded on the supporting structures, especially in the situation of combining the mini dental implant as a complementary supporting structure at the mandibular distal extension ridge. However, to the best of our knowledge, there are currently no studies addressing the possibility of applying a mini dental implant as a supporting component in the distal extension ridge. For these reasons, the present research aims to investigate the effect of different clasp designs on the stress distribution pattern and the maximum von Mises stress on the supporting structures as well as to evaluate the suitability of the mini dental implants in conjunction with mandibular distal extension RPDs as the complementary supporting component.

In the present study, the 3D finite element analysis (3D FEA), which is a well-accepted technique for theoretical calculating of stress distribution within a complex model, was performed to investigate the stress distribution pattern and the maximum von Mises stress on supporting structures between the CRPDs and the IARPDs. The results from our study could be valuable for developing the concept of distal extension base RPD designs that focused on preserving the integrity of the remaining oral structures, providing great satisfaction in function and the high survival rate of RPD supporting structures including the assisted dental implants on the free-end saddle areas.

## 2. Materials and Method

**Figure 1:**
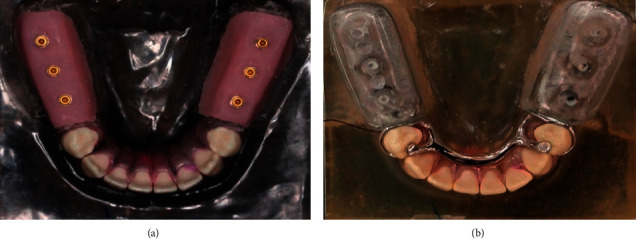
(a) The mandibular model with preformed acrylic resin teeth, the polyvinyl siloxane-simulated PDL, and gingival tissue on edentulous ridges. (b) RPD metal framework for distal extension RPD with bilateral acrylic resin bases on the edentulous areas.

Two identical chemical-cured resin acrylic mandibular models with preformed resin teeth (PE-ANA002®; Nissin, Kyoto, Japan) from 34 to 44 and bilateral distal extension edentulous ridges were fabricated. For the model assigned for FEA model fabrication, the polyvinyl siloxane (GI Mask Automix, Coltene, Madrid, Spain) was used to simulate the 2 mm gingival mucosa covered on edentulous ridges with an average of 0.3 mm periodontal ligament surrounded on the root surface of abutment teeth as described by a previous study (Figure 1(a)) [22]. The second model was prepared as a master model for framework fabrication. The cobalt-chrome-molybdenum RPD framework composed of a lingual bar, mesio-occlusal rest, distal proximal plate, and Akers clasps on both first premolar abutments with distal extension acrylic bases was performed in a standard manner (Figure 1(b)).

**Figure 2:**
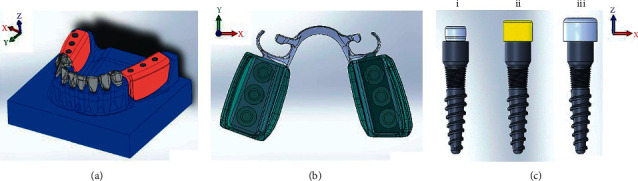
The separately prepared components of the FEA model: (a) the mandible with bilateral distal extension ridges, (b) the distal extension RPD metal framework with acrylic bases, and (c) mini dental implant connected with equator attachments.

A scanner (3Shape lab scanners D850 Copyright ^©^ 3Shape A/S., Denmark) and the intraoral scanner (http://www.dentalproductsreport.com/dental/article/cda-2012-audio-interview-3shape-about-trios-digital-impression-systemTRIOS® intraoral scanner Copyright ^©^ 3Shape A/S., Denmark) were utilized for converting the acrylic model and denture to digital files. The digital file of mini dental implant connected with equator attachment (Figure 2(c), (i)), the mini dental implant connected with equator attachment and silicone cap (Figure 2(c), ii), and the mini dental implant connected with equator attachment, silicone cap, and metal housing (Figure 2(c), iii) were simulated (PWplus CO., LTD., Nakhon Pathom, Thailand). The TRIOS^®^ Orthodontics software was used to process the digital files into STL format. The prepared components are presented in Figure 2.

**Figure 3:**
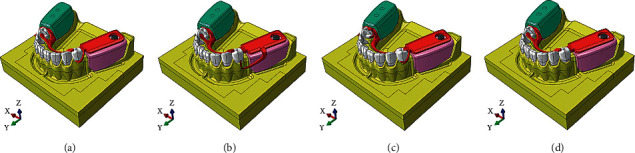
The 3D FEA models of bilateral distal extension base mandibular arch with the RPD metal framework with the different designs of retentive component and the acrylic denture bases on edentulous areas: (a) the model with the RPA clasps, (b) the model with the RPI clasps, (c) the model with the Akers cast clasps, and (d) the disto-occlusal rest seats with no retentive components as control.

The separately prepared components were assembled with SolidWorks 2015 (Dassault System, France) and saved in SLDPRT format to form the drafted 3D FEA models (Figure 3). A duplicated copy of the data was converted into parasolid XT format for further analysis in Abaqus 6.13 (SIMULIA, Providence, RI, USA). The models of conventional RPD with different framework designs are presented in Figure 3.

**Figure 4:**
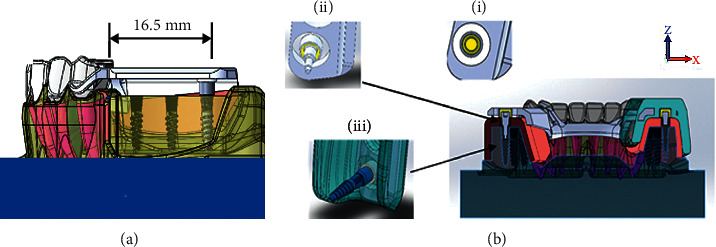
The relationship among the mini dental implant, equator attachment system, and the prepared model with RPD framework with acrylic base on edentulous areas: (a) the placement of mini dental implant at 16.5 mm distal to the distal surface of the last abutment tooth on both sides of edentulous ridges and (b) the silicon cap within the acrylic base attached on the RPD framework (i), the connection between the equator attachment and silicon cap within the acrylic base (ii), and the connection of the mini dental implant to the equator attachment system within acrylic base (iii).

The 3D FEA models for the mini dental implant-assisted RPD group were created by replacing the areas of the second molars on both sides of conventional RPD models with simulated cylindrical, noncontacting, peri-implant bone combining with mini dental implants connected with silicone cap and equator attachment. The relationship between mini dental implant and equator attachment to the mandibular model is shown in Figure 4.

**Figure 5:**
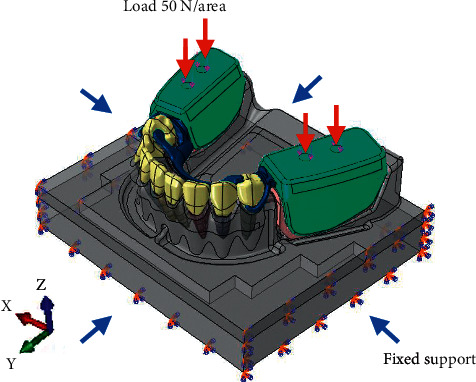
Full isotropic view of the FE model with loading and boundary condition.

The FEA model components including the mandible with distal extension ridges, RPD metal framework with acrylic base, and mini dental implant combined with equator attachment were divided into small tetrahedral elements with 10 nodes each. A total of 1,455,395 elements, 1,555,146 elements, 1,431,871 elements, and 1,433,607 elements were used for models with mini-implant placements Gr2, Gr4, Gr6, and Gr7, respectively. A full isotropic view of the FE model with loading and boundary conditions were assigned (Figure 5). The implant was designed to be bonded to the cancellous and cortical bone to simulate the complete osteointegration. The meshes for the root were surrounded by meshes of the periodontal ligament that were 0.3 mm thick. The assemble of mini dental implants and peri-implants was meshed as tetrahedral elements. Tie contacts were applied when 100% osseointegration were assumed. To eliminate the movement effects, which were not considered in this study, the contact between clasps and abutment teeth was set as a frictional contact with the friction coefficient of 1. For models with mini dental implant placement, the simulation of the cylindrical, noncontacting, peri-implant bone was done by subtracting from the mandible model using Abaqus software. The mechanical properties of the different component of the FEA model are exhibited in Table 1.

A total of 200 N was applied on the distal extension base. To evenly simulate the vertical occlusal load in the maximum intercuspal position, the vertical loading of 50 N with the loading area of 1.5 mm^2^ was generated on the acrylic base at the distance of 9 and 12 mm from the distal surface of the last left and right abutment teeth of the mandibular model to simulate the point contacts on opposing flat occlusal surfaces (Figure 5). To control the applied force as recommended in clinical scenario for distal extension removable partial denture [29], only bilateral vertical loading was applied to control occlusal force in vertical direction.

The measurement from the strain gauge model under the same conditions was utilized to validate the 3D FEA model at the surface area of the FEA model and the strain gauges attached. The results obtained from both FEA and strain gauge measurements were correlated within an error margin of less than 10% for most of the gauges were obtained.

The prepared 3D FEA models were divided into seven groups according to the clasp design as presented on Table 2.

The effect of vertical load application on the edentulous ridge, root surface of abutment teeth, mini dental implant, and surrounding bone were analyzed. Descriptive statistics were utilized for analyzing the stress distribution pattern and maximum von Mises stress on the edentulous ridges, abutment teeth, peri-implant bone, and mini dental implant. The average hydrostatic pressure of the periodontium around the abutment teeth and the mucosal edentulous areas were also evaluated, and comparisons were conducted between the same and different clasp designs in the CRPD and IARPD groups.

## 3. Results

**Figure 6:**
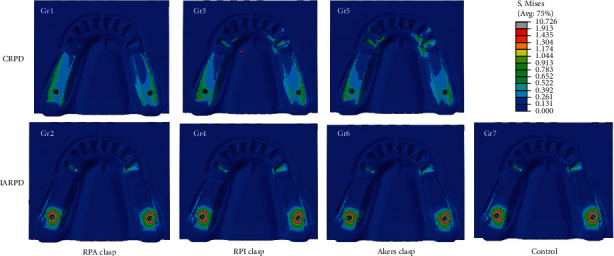
Stress distribution patterns on the edentulous areas of CRPD and IARPD models with various retentive clasp designs. The variation in retentive clasp designs of CRPDs and IARPDs provided less effect on the stress distribution patterns in both groups.

The stress distribution pattern on the edentulous ridge of the CRPD and IARPD with various clasp designs is presented in Figure 6. The approximate pattern of stress distribution was observed on the edentulous ridges in all groups of the conventional tooth-tissue-supported RPD. However, the variation in retentive clasp designs in the CRPDs generated different stress distribution patterns on alveolar bone around the abutment teeth. The expression of stress distribution patterns on alveolar bone at canine and first premolar abutments was observed on the models with Aker and RPI clasps, while the model with RPA clasp did not show stress on alveolar bone sockets. When mini dental implants were installed on each side of edentulous ridge, the obvious pattern of the stress concentrated around the mini dental implants placed distally on the distal extension ridges, as observed in all groups of IARPD model. The same result was also exhibited on the IARPD without retentive component (control group).

**Figure 7:**
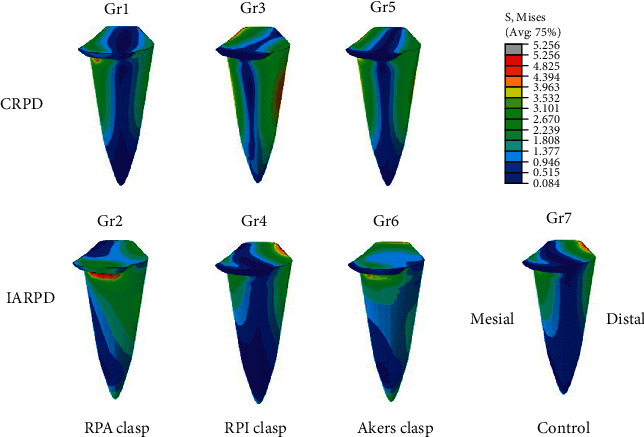
The stress distribution pattern on the abutment root surfaces following the application of a total 200 N vertical loading at distal extension ridges of the CRPD and IARPD with various retentive clasp designs.

For the stress on the abutment root surfaces, the highest maximum von Mises stress area on the distobuccal surface at the cervical region was found on the abutment root surfaces of CRPD and IARPD with suprabulge clasp design (Gr1, Gr2, Gr5 and Gr6) as exhibited in Figure 7. For the CRPD model with infrabulge clasp design (Gr3), the highest maximum von Mises stress area was observed at the cervical and middle part of root surface. For the models with bilateral placement of mini dental implants on the edentulous ridges, the highest maximum von Mises stress was obviously seen around the mini dental implants in all groups regardless of the different designs of the retentive component.

**Figure 8:**
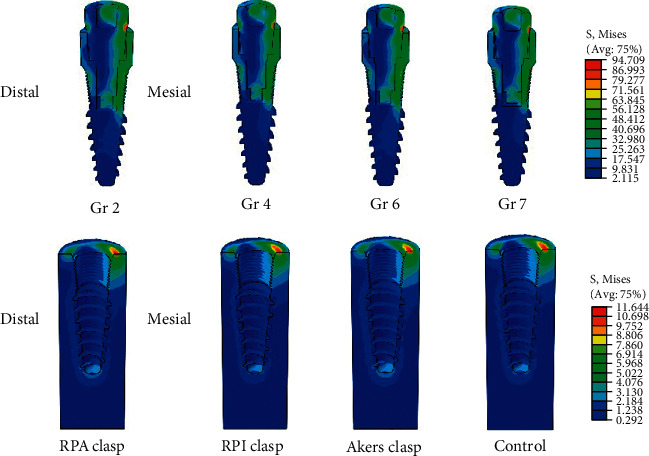
The stress distribution pattern on the mini dental implant and the peri-implant bone when applied vertical load of 200 N on the edentulous ridge areas of IARPD with different retentive clasp designs.

Focusing on the stress distribution pattern generated on peri-implant bone and mini dental implant following the application of a total 200 N vertical loading, the same patterns were observed in all groups of IARPD regardless of the design of retentive component as presented in Figure 8. The higher stress distribution on mesial at alveolar bone crest was found on peri-implant bone in all models. The stress-concentrated areas were found on the mesial at the level of the most occlusal contact between the attachment and the implant body.

The highest von Mises stress values of each model are summarized in Table 3. The different design of retentive component caused different maximum von Mises stress on RPD supporting structures (abutment root surfaces, peri-implant bone, and mini dental implant). The lowest value was observed when the RPI clasp was selected as the retentive component. For models with RPA and Akers clasp design, not significantly different von Mises stress values were observed in each supporting structure. The IARPD with Akers clasp design showed the highest maximum von Mises stress on supporting structures followed by IARPD with RPA and RPI clasp, respectively.

CRPD = conventional RPD design, IARPD = implant-assisted RPD design.

The fluid-induced stress on the periodontal ligament and mucosal tissue covered on edentulous ridges known as mechanically hydrostatic pressure in all groups of both the CRPD and the IARPD was in approximate. A slightly higher hydrostatic pressure on the mucosal tissue covered on the edentulous ridges was observed in model with RPA clasp and the models without retentive component (control) as shown in Table 4. The comparison between the maximum von Mises stress found on each component and its elastic modulus is presented in Table 5.

## 4. Discussion

The stress distribution pattern following mastication and other oral functions plays a significant influence on the integrity of the supporting structures as well as the longevity of dental prostheses. The compatibility of the stress and the bearing capacity of the supporting structures is one of the fundamental requirements for developing the appropriate treatment plan or designing of the restorations that harmonizes in the aspect of the biomechanics and the anatomical structures of each patient. In our present study, the results of the stress distribution pattern and maximum von Mises stress value can be classified and discussed as follows.

### 4.1. The Effect of Clasp Design on Stress Distribution Pattern and Maximum von Mises Stress on the Supporting Structures

Our present study indicated that the different designs of the retentive component affected the pattern of stress distribution and maximum von Mises stress value on the supporting structures. These data conformed to the clinical study by McCartney [30] that addressed the direct effect of the retentive clasp design to the direction and load distribution on the abutment teeth and supporting structures. In the present study, the least maximum von Mises stress value on the abutment root surfaces had been observed in the model with the stress breaker clasp design (RPI clasp). This design allows the disengagement of the retentive component from the undercut area on abutment teeth when the denture base moved downward to the mucosa during functions. This movement resulted in reduction of stress and torque on supporting structures especially in mandibular distal extension base RPDs. Due to this advantage, the retentive clasp design with a stress breaker concept has been recommended for use to compensate the effect of the difference in vertical movement between abutment teeth and edentulous ridge in distal extension base RPDs [9, 11, 13].

Moreover, the design of RPD clasp also influenced the maximum von Mises stress value on the supporting components. The present study found that the suprabulge clasp design (RPA and Akers clasp) provided the higher maximum von Mises stress on the root surfaces of the abutment teeth in both the conventional and implant-assisted RPD models. This result is consistent with the study of Taylor et al. [31] that suggested that the circumferential casting clasp is more likely to generate higher torque to the abutment teeth than the retentive arm in I-bar shape. These data are also in agreement with study by Aoda et al. [32] that reported the highest load on the abutment teeth adjacent to the Kennedy class II edentulous arch when Akers clasp had been applied as a retentive component.

When focusing on the location of the maximum von Mises stress on abutment root surface, the areas of the highest stress were observed distally at the level of the cervical third of the abutment root surfaces in all CRPD groups. However, the lingual shifting of the maximum stress-accumulated area was found in the infrabulge clasp designs of the IARPD group. In overview, the placement of MDIs bilaterally at the distal extension bases resulted in alteration of vertical movement caused by the different resiliency between abutment teeth and edentulous ridges [33]. The formation of the maximum stress concentrated on the distal of the abutment root surfaces next to the distal extension ridge had the possibility of being harmful to abutment teeth and surrounding bone, as too much stress might lead to the horizontal bone resorption and the bodily movements of the abutment teeth, which results in disrupting the dental alignment and normal occlusal. Conversely, if the clasp design allowed the stress to form mesially, there could be less undesirable outcome due to the bracing action from the other teeth anteriorly to the abutment [9].

### 4.2. The Effect of Rest Position on the Stress Distribution Pattern and Maximum von Mises Stress on the Supporting Structures

In conventional distal extension base RPDs, the position of occlusal rest on abutment teeth next to the distal extension ridges is one of the significant factors that influenced the stress distribution pattern on the supporting structures [34]. The placement of occlusal rest on mesial marginal ridge on the last abutment teeth next to the distal extension ridges resulted in the formation of fulcrum line anteriorly to the tip of retentive clasp that led to the alteration of the lever situation from type I to type II. This design does not only decrease the torque on abutment teeth but also reduces the necessity of incorporating an indirect retainer in the metal framework of distal extension base RPDs for preventing the denture base lifting during function [35].

A coincidence was found in the present study, where a smaller value of maximum von Mises stress in implant-assisted RPD with RPA clasp design (Gr2) compared with the Akers clasp with distal rest seat on the last abutment teeth (Gr6) was observed. It is possible that the design of disto-occlusal rest on mesial marginal ridge of the last abutment teeth next to the edentulous ridge prevents the distal displacement of the denture due to the masticatory load, resulting in a lesser maximum von Mises stress at the distal surface of the MDI and peri-implant bone [36]. However, a finite element analysis of a simulated CRPD by Muraki H. et al. [37] that compared the mobility and stress on abutment teeth of the RPD framework with mesial or distal occlusal rest found that a distal occlusal rest caused more movements, generating up to 0.350 MPa of stress in the PDL. The stress occurring around the alveolar bone of the abutment teeth is less than the physiologic limit, which is 24.5166 MPa (250 kg/cm^2^) at the cervical and 2.4517 MPa (25 kg/cm^2^) at the apical bone [38]. Considering our data, the average hydrostatic pressure on all CRPD and IARPD models (Table 4) was less than the value reported by Muraki et al. From this reason, it could be assumed that the stress distribution pattern and maximum von Mises stress generated on supporting structures because of the different retentive clasp designs are in the range of the physiological limit that could not initiate the process of alveolar bone resorption.

### 4.3. The Average Hydrostatic Pressure in the Periodontium of the Abutment Teeth and Mucosal Edentulous Areas

The periodontium around the root surfaces of the abutment teeth and gingival tissue covered on edentulous ridges are easily subjected to displacement and deformation following load application. To evaluate the response of soft tissues following the load application, the average hydrostatic pressure is one of the parameters that can be effectively utilized. Chen et al. [25] found that the soft tissues might be ischemic if they had loaded with forces greater than their physiologic limit for a long period of time. This compromised blood supply situation leads to the stimulation of the inflammatory system, which related to the progression of alveolar bone or root resorption process. Hohmann et al. [39] highlighted that the progression of the abutment root resorption will start when the average hydrostatic pressure exceeds 4.7 kPa. Furthermore, Liao et al. [40] hypothesizes that if the hydrostatic pressure exceeds the systolic blood pressure of 120 mmHg (16 kPa), the tissues in that area will lose its vascularization and the initiation of inflammation process will occur. In our present study, the average value of the hydrostatic pressure in all groups exceeded the limited state mentioned in the previous studies. This can be explained by the difference in defining the properties and characteristic of periodontium and gingival tissue in FEA models. For the present study, the periodontium is defined as an isotropic linear elastic material with homogeneous contact, while the previous studies defined it as an anisotropic nonlinear elastic material with heterogeneous contact [23]. Therefore, the average hydrostatic pressure in the current study is only comparable between the groups in this study, which were analyzed under the same conditions.

The study by Matsudate et al. (2016) [41] found the highest stress on the periodontium of the abutment teeth and the least stress on the edentulous areas occurred when the distally implant placement had been utilized. They stated that the normal-sized implant installed at the distal part of the distal extension ridge functioned as an assisted supporting structure for distal extension RPD. In the current study, the average hydrostatic pressure of the IARPD group was in approximation with the CRPD group. It can be inferred that a single distally mini-implant placement bilaterally on each side of the distal extension edentulous areas in the present study did not mainly function as the supporting structure but rather increased the denture retention and stability, which plays a significant role in the reduction of the denture displacement during oral function.

Within the limitations of the current study, the results can only predict the behavior of RPD supporting structures under certain circumstances. The FEA was used in the current study to assess the effect of the mini dental implant-assisted RPD with different retentive clasp designs on the stress distribution pattern and maximum von Mises stress in denture supporting structures. The limitations in this present study are the fact that the geometry of the models might not be clinically accurate, the mechanical properties defined might differ from the exact properties of each tissue, there was absence of the reactions between different materials contacting, and the load was only applied vertically. The results from the current study are limited to the very controlled virtual simulation and cannot refer to the situation where changing and multidirectional forces and stress are presented, as in the oral cavity. Further studies could investigate the effects of loading in other directions and more accurately replicate the different clinical conditions such as the unilateral loading found in the mutually protected occlusion or the bilateral loading in bilateral balanced occlusion. Moreover, a prospective study should be processed and compared the results with the FEA study.

## 5. Conclusion

Based on the results from the numerical analysis, the implementation of the distal extension base RPD design by the incorporation of the infrabulge retentive clasp with mesio-occlusal rests and assisted on each side of distal extension base with mini dental implant attached with locator attachment resulted in a reduction of stress on the supporting structures. The proper design of retentive clasp for implant-assisted RPDs should not be based only on the amount of the retentive force but also be concerned about the capability of each retentive clasp design for preserving the supporting structures in the long term.

## Figures and Tables

**Table 1:** Material properties.

	Elastic modulus (MPa)	Poisson's ratio
Mucosa [25]	0.680	0.45
Periodontal ligament [12]	0.689	0.49
Mandibular bone [12]	4,042.5	0.30
Tooth [12]	41,000	0.30
RPD framework (Co-Cr) [26]	220,000	0.30
Acrylic resin [26]	2,200	0.31
Implant (Ti) [27, 28]	110,000	0.35

**Table 2:** Finite element models.

Group	Design	No. of nodes	No. of elements
1	Free-end RPD with RPA clasp	740,460	1,449,435
2	Mini dental implant-assisted RPD with RPA clasp	750,926	1,455,395
3	Free-end RPD with RPI clasp	581,119	1,544,170
4	Mini dental implant-assisted RPD with RPI clasp	592,891	1,555,146
5	Free-end RPD with Akers clasp and distal rests	690,364	1,421,038
6	Mini dental implant-assisted RPD with Akers clasp and distal rests	702,106	1,431,871
7	Free-end RPD with mesial rests and no retentive components	746,329	1,433,607

**Table 3:** Maximum von Mises stress values (MPa) in each component.

Model		Maximum von Mises stress (MPa)			
		RPA	RPI	Akers	Control
Abutment root surface of 34	CRPD	17.55	6.32	16.34	-
	IARPD	11.14	2.54	10.35	2.36
Abutment root surface of 44	CRPD	18.28	6.28	19.35	-
	IARPD	10.71	2.03	11.36	2.06
Mini dental implant at area 37	CRPD	-	-	-	-
	IARPD	19.02	14.22	22.63	14.12
Mini dental implant at area 47	CRPD	-	-	-	-
	IARPD	18.31	15.78	22.66	15.52
Peri-implant bone at area 37	CRPD	-	-	-	-
	IARPD	2.61	1.60	3.00	1.60
Peri-implant bone at area 47	CRPD	-	-	-	-
	IARPD	2.38	1.80	3.20	1.76

**Table 4:** Average hydrostatic pressure of the periodontium and mucosal tissue on the edentulous areas (MPa).

Group	PDL 34	PDL 44	Left mucosa (Q3)	Right mucosa (Q4)
1	0.0257	0.0300	0.0681	0.0549
2	0.0257	0.0300	0.0681	0.0703
3	0.0257	0.0300	0.0590	0.0568
4	0.0257	0.0300	0.0590	0.0568
5	0.0257	0.0300	0.0590	0.0568
6	0.0257	0.0300	0.0590	0.0568
7	0.0257	0.0300	0.0681	0.0701

**Table 5:** Maximum von Mises stress compared to the elastic modulus of the materials (MPa).

Component	Elastic modulus	M1	M2	M3	M4	M5	M6	M7
Peri-implant bone	4,042.5	1.18	2.61	1.18	1.80	1.17	3.20	1.76
Tooth	41,000	340.40	237.00	138.60	79.99	105.60	93.36	81.54
RPD framework (Co-Cr)	220,000	284.80	202.00	278.00	240.90	110.60	53.93	169.60
Acrylic resin	2,200	24.77	24.83	7.97	4.56	25.14	13.42	35.50
Implant (Ti)	110,000	3.99	19.74	4.19	15.78	3.97	22.66	15.52

## Data Availability

The data used to support the findings of this study are available from the corresponding author upon request.
